# Dialectical behavior therapy for justice-involved veterans: randomized clinical trial protocol comparing treatment approaches for veterans with criminal-legal involvement

**DOI:** 10.1186/s40359-026-04749-2

**Published:** 2026-06-24

**Authors:** Emily R. Edwards, Ryan Holliday, Sharon Alter, Daniel Blonigen, Suzanne E. Decker, Ariana Dichiara, Gabriella Epshteyn, Jeri Forster, Anthony J. Fortuna, Michele Galietta, Nathan A. Kimbrel, Matthew Stimmel, Suzanne Thomas, Jack Tsai, Marianne Goodman

**Affiliations:** 1 VISN 2 Mental Illness Research, Education, and Clinical Center, US Department of Veterans Affairs, Bronx, NY USA; 2 Department of Psychiatry, Icahn School of Medicine at Mount Sinai, New York, NY USA; 3 VA Pacific Islands Health Care, Honolulu, HI USA; 4 VA Eastern Colorado Health Care System, Aurora, CO USA; 5 Department of Psychiatry, University of Colorado Denver - Anschutz Medical Campus, Aurora, CO USA; 6 Department of Physical Medicine & Rehabilitation, University of Colorado Denver - Anschutz Medical Campus, Aurora, CO USA; 7 Center for Innovation to Implementation, VA Palo Alto Health Care System, Palo Alto, CA USA; 8 Department of Psychiatry and Behavioral Sciences, Stanford University School of Medicine, Stanford, CA USA; 9 Department of Psychiatry, Yale School of Medicine, New Haven, CT USA; 10 VISN 1 Mental Illness Research, Education, and Clinical Center, US Department of Veterans Affairs, West Haven, CT USA; 11 Department of Psychology, University of Rhode Island, Kingston, RI USA; 12 Rocky Mountain Mental Illness Research, Education, and Clinical Center for Suicide Prevention, US Department of Veterans Affairs, Aurora, CO USA; 13 Department of Psychology, Fordham University, Bronx, NY USA; 14 Department of Psychology, John Jay College of Criminal Justice, New York, NY USA; 15 Mid-Atlantic Mental Illness, Research, Education, and Clinical Center, US Department of Veterans Affairs, Durham, NC USA; 16 Department of Psychiatry and Behavioral Sciences, Duke University School of Medicine, Durham, NC USA; 17 Veterans Justice Programs, US Department of Veterans Affairs, Washington, DC USA; 18 National Center On Homelessness Among Veterans, US Department of Veterans Affairs, Washington, DC USA; 19 School of Public Health, University of Texas Health Science Center, Houston, TX USA

**Keywords:** Veteran, Criminal justice, Legal, Clinical trial, Protocol, Suicide

## Abstract

Criminal-legal involvement is a commonly overlooked risk factor for suicide, preceding up to 30% of suicide deaths in the United States (U.S.). Interventions to reduce risk for suicide and/or criminal-legal involvement, however, rarely address these outcomes concurrently. This paper describes a recently developed intervention, Dialectical Behavior Therapy for Justice-Involved Veterans (DBT-J), that was specifically designed to address concurrent risk of criminal-legal involvement and suicide among at-risk U.S. military Veterans. A protocol for a multisite randomized clinical trial to evaluate the efficacy of this intervention is also outlined (clinicaltrials.gov ID: NCT05974553, registered July 26, 2023). Development and evaluation of this intervention highlight necessary considerations around culture and clinical needs of the presenting population, suicide risk assessment and management strategies, and treatment engagement. Each of these considerations can be readily applied to development, evaluation, and implementation of programs for similarly at-risk populations.

**Supplementary Information:**

The online version contains supplementary material available at https://doi.org/10.1186/s40359-026-04749-2.

Criminal-legal involvement is a salient yet frequently under-assessed risk factor for suicide. In 2019, 27.2% of suicide deaths in the United States (U.S.) occurred among individuals who had been incarcerated within the previous two years [1]. Results of a recent meta-analysis also suggest risk for suicide is more strongly associated with criminal-legal involvement than many other social determinants of health, including exposure to suicide mortality, firearm access, and occupational stress [2]. Risk for suicide is also particularly elevated among persons with co-occurring difficulties, such as mental health concerns or housing instability [3, 4]. Preventing suicide therefore requires addressing the criminal-legal involvement and co-occurring difficulties of at-risk persons. This manuscript describes an intervention to decrease criminogenic and suicide risk among U.S. military Veterans experiencing criminal-legal involvement (VCLI) and outlines a corresponding clinical trial to evaluate the efficacy of this intervention. Particular attention is paid to considerations around culture and clinical needs of the presenting population, suicide risk assessment and management, and treatment engagement strategies required in development and evaluation of interventions for individuals at concurrent risk for criminal-legal involvement and suicide.

## Veterans with criminal-legal involvement

VCLI are a high-risk, high-need population overrepresented in behavioral healthcare settings within the U.S. Department of Veterans Affairs (VA), a federal entity that provides healthcare, social services (e.g., housing support), benefits (e.g., financial compensation for service-related injuries), and funeral services to eligible military Veterans (VA; [5]. In 2023, the suicide rate for VCLI was approximately 145 per 100,000, over four times that of Veterans overall and over eight times that of civilian adults [6]. Results of a recent meta-analysis suggest 81% of VCLI experience a mental health condition and 69% experience a substance use disorder; rates of mood, trauma-related, and personality disorders appear particularly overrepresented in this population [7]. In comparison to Veterans without a history of criminal-legal involvement, VCLI are also more likely to engage in suicidal, violent, and otherwise aggressive behaviors and to experience homelessness, occupational instability, and poor community integration [8–11]. These challenges may contribute to difficulties with sustained service engagement by increasing transience, introducing demands for complex care coordination, and prompting crisis intervention that can interfere with care continuity (e.g., inpatient hospital stays).

To support VCLI, VA offers programming through Veterans Justice Programs (VJP) to connect Veterans with VA-based health care and case management services, including mental health and substance use disorder treatment, primary care, housing support, and other identified areas of need [12, 13]. Nevertheless, when any services are provided in isolation, they are likely insufficient in reducing risk for criminal-legal involvement and addressing the range of difficulties commonly faced by VCLIs [14, 15]. Forensic-oriented interventions that specifically target risk factors for criminal-legal involvement (e.g., antisocial attitudes and behaviors) are likely necessary. However, a survey of VJP specialists suggests numerous barriers to implementing such interventions, including patient perceptions of forensic programming as lengthy, intensive, and unappealing to internal motivations and provider biases against Veterans who engage in antisocial behaviors [16]. Consistent with this, recent efforts to implement and evaluate Moral Reconation Therapy [17] – an evidence-based intervention to decrease criminogenic thinking – in VA settings faced notable difficulties with patient engagement and staff adoption [18, 19].

Considering these barriers, effective clinical interventions for VCLI will likely require programming to be brief, appealing to VCLI’s internal motivations, and relevant to behavioral health providers. Such intervention would also need to balance efforts across a range of treatment targets, including risk for criminal-legal involvement, risk for suicide, behavioral health concerns, and other forms of psychosocial stress (e.g., housing instability, financial insecurity, etc.).

## Dialectical behavior therapy for justice-involved veterans

Dialectical Behavior Therapy for Justice-Involved Veterans (DBT-J; [20] was developed with consideration of the presenting needs of VCLI and identified barriers to implementing forensic-oriented programming in VA settings. It combines elements from leading treatment modalities in offender rehabilitation (Risk-Need-Responsivity Model [RNR]; [21], suicide and mental health (Dialectical Behavior Therapy [DBT]; [22], and forensic case management (Socioenvironmental Disadvantage Model [SDM]; [14] to offer an integrative, manualized approach to simultaneously address VCLI’s criminogenic, behavioral health, and case management needs. The structure and content of DBT-J was also informed by feedback from experts in intervention development for persons at risk for suicide and criminal-legal involvement, VJP specialists, and VCLI themselves.

### Treatment structure

DBT-J provides 1–1.5 h of intervention per week for 16 weeks through group therapy, individual case management, measurement-based care, and provider participation in a specialized consultation team. The DBT-J program structure is consistent with recent research suggesting DBT-based interventions that are abbreviated and/or comprise only group therapy, case management, and consultation team typically yield outcomes comparable to the 12-month, comprehensive DBT-model [23, 24], even in the treatment of legally-involved persons [25]. The combination of group therapy and case management interventions allows DBT-J to be both standardized and individualized to each Veteran’s unique needs. All elements of DBT-J are provided via telehealth, thereby facilitating sustained engagement even while navigating difficult life circumstances, such as transportation barriers, housing instability, and admission to residential treatment.

#### Group therapy

The group therapy component of DBT-J promotes development, strengthening, and generalization of behavioral skills to effectively manage emotions, behaviors, and relationships underlying criminogenic risk and behavioral health difficulties. This component progresses through six modules – observing limits, foundational mindfulness, emotion regulation, goal directedness, interpersonal relationships, and relapse prevention. See Table 1. The included skills draw from RNR- and DBT-based interventions to maximize relevance of group content to VCLI needs. For example, given previous research implicating poor emotion processing and regulation – particularly in response to trauma – as underlying offending behavior [26, 27], the emotion regulation module draws heavily from DBT to teach foundational skills in understanding, processing, and managing emotional reactions and behavior. Further, given common difficulties in empathy and interpersonal connection among legally-involved persons [28, 29], the interpersonal relationships module includes a *mindfulness of others* skill through which participants learn step-by-step strategies for validation and building emotional closeness with others. Each group session includes: (a) completion of a brief mindfulness exercise (5 min); (b) review of homework from the previous week (20 min); (c) introduction and in-session practice of a new skill (30 min); and (d) assigning of between-session homework to promote generalization of skills to real-life experiences (5 min).

**Table 1:** Correspondence between DBT-J content and treatment targets

Skills Group Schedule	Corresponding Treatment Target												
	Criminogenic Needs							Behavioral Health Needs					Socioenvironmental Needs
	Antisocial Personality	Antisocial Attitudes	Antisocial Peers	Substance Use	Educational / Vocational Difficulties	Family Difficulties	Lack of Prosocial Activities	Suicide Risk	Mood Disruption	Emotion Dysregulation	Interpersonal Difficulty	Executive Functioning Disruption	Case Management Difficulties
Observing Limits	X	X	X	X		X					X		
Mindfulness	X			X		X		X	X	X	X	X	
Dialectical Thinking	X			X		X		X	X	X	X	X	
Mindfulness of Self				X				X	X	X			
Emotional Schemas		X		X				X	X	X			
Thinking Errors	X	X		X				X	X	X			
Check the Facts & Opposite Action				X				X	X	X			
Self-Soothe & TIPP								X	X	X			
Values Clarification	X	X	X	X	X	X	X			X	X	X	X
Mindfulness of Goals	X		X	X	X	X	X			X	X	X	X
Problem Solving	X				X	X	X	X		X	X	X	X
Distress Tolerance & Radical Acceptance	X			X	X			X	X	X			X
Assertiveness	X		X			X					X		X
Mindfulness of Others	X	X	X			X					X		
Making Prosocial Relationships			X								X		
Relapse Prevention	X			X			X	X	X				

#### Case management

Case management sessions, led by the same provider leading the group therapy portion of DBT-J, are held every other week for up to 30 min to assist in coordinating connection to and care between the treatment team, legal services, and social services within the VA system and/or VCLI’s community. For example, for VCLI with active substance use, sessions may facilitate connection to local 12-step programs to support efforts toward sobriety. For VCLI facing financial difficulties, sessions may include referral to VA benefits advisors. Where possible, providers coach clients through applying skills learned in group therapy sessions (e.g., problem solving) to resolve case-management difficulties. Motivational interviewing strategies are also used throughout these sessions to promote sustained connection to care. Informed by SDM, case management is tailored to the client’s unique needs and balances foci on resolving factors contributing to criminogenic risk (e.g., substance use) with those prioritized by the Veteran (e.g., financial instability).

#### Assessment

Periodic assessments throughout the course of DBT-J participation allow for measurement-based care, consistently shown to improve treatment effectiveness [30]. Assessments include, for example, previously validated measures of criminogenic risk (e.g., The Level of Service / Case Management Inventory; [31], psychosocial functioning (Inventory of Psychosocial Functioning; [32], and suicide risk (e.g., Beck Scale for Suicidal Ideation; [33]. Assessment results are shared with participating VCLI during individual case management sessions, documented in Veterans’ electronic medical records, and used to guide collaborative treatment and case management planning.

#### Consultation team

The final treatment component, provider involvement in a weekly consultation team meeting, is considered standard for traditional DBT programs [22]. These meetings are attended by providers only; VCLI do not attend or participate in consultation team meetings. Consultation team meetings provide a forum for providers to troubleshoot barriers to care, support one another, promote adherence to the treatment model, and protect against provider burnout [34].

### Early evidence of efficacy

Two small scale clinical trials have evaluated the feasibility, acceptability, and preliminary efficacy of DBT-J [20, 35]. Across both studies, results suggested DBT-J is feasible and acceptable, with strong attendance, high engagement, and positive qualitative feedback about the program from VCLIs and referring providers alike. Participants reflected the great heterogeneity of the broader VCLI population. Collapsing across both studies, nearly half of participants had a personality disorder, two-thirds had a current substance use disorder, and over three-quarters had another form of mental health condition (i.e., mood, psychotic, anxiety, trauma-related, and/or intermittent explosive disorder). Consistent with RNR principles suggesting highest-intensity services should be reserved for persons at higher risk for criminogenic behavior [21], collapsing across trials, 82% were at moderate or high risk of criminal behavior upon admission, and none were at high risk upon program completion [20, 35].

Qualitative feedback from the first of these trials suggested the program’s relatively brief duration and balanced focus on criminogenic and non-criminogenic needs appealed to VCLI’s internal motivations and encouraged strong engagement, whereas it’s similarity to traditional DBT skills groups and partial focus on behavioral health appealed to VA providers, treatment courts, and other potential referral sources [20]. In the second trial, those who completed DBT-J experienced statistically significant improvements in risk for criminal behavior from pre- to post-treatment, and these gains were maintained at one-month follow-up [35].

Participants in these studies provided pivotal feedback to increase the cultural responsiveness of DBT-J for a U.S. military Veteran population. For example, VCLI offered culturally relevant metaphors to support skill presentation (e.g., drawing parallels between emotion mind-reasonable mind distinctions and off duty-on duty mindsets during military service), highlighted the potential contributions of prior service to current experiences (e.g., military culture as a potential contributor to emotional schemas and thinking habits), and suggested adaptations to the clinical style of group leaders to better mirror interpersonal styles common within military contexts (e.g., increased use of irreverence).

### DBT-J and suicide risk

The structure and content of DBT-J directly targets overlapping risk factors for antisocial behavior and suicide by: (a) building and strengthening behavioral skills for observing limits, mindfulness, emotion regulation, goal directedness, interpersonal effectiveness, and relapse prevention; and (b) facilitating connection to services and resources that reinforce use of adaptive skills. In early DBT-J trials, one-third of participating Veterans had a history of attempting suicide, and one-third were experiencing suicidal ideation upon program admission [20, 35]. Although analyses were underpowered to detect statistically significant effects, Veterans who completed the program experienced, on average, moderately sized (*d* = 0.30–0.88) improvements in multiple suicide risk factors across the course of treatment, including substance use, psychological distress, and quality of life [35].

DBT-J providers utilize a formal suicide risk assessment and management protocol designed for Veteran samples and VA contexts [36] to facilitate ongoing risk monitoring and efficient, therapeutic responses to acute changes in VCLI suicide risk. Comprehensive suicide risk assessments prior to program entry provide an initial conceptualization of VCLI acute and chronic risk for suicide (i.e., immediate risk and propensity toward increases in immediate risk, respectively). Ongoing monitoring of risk and protective factors is then used throughout the course of the VCLI’s participation in DBT-J to guide suicide risk assessment and management. Notable changes in risk and/or protective factors (e.g., increase in psychosocial stress, decrease in social support) prompt more structured suicide risk assessment and management strategies as necessary to promote VCLI safety. Various VA-based supports, such as those offered by the Veterans Crisis Line, VA Suicide Prevention Coordinators, and other behavioral health providers, are also integrated into suicide risk management efforts to promote effective, sustained risk mitigation.

### DBT-J engagement strategies

Wide ranging psychosocial needs, relational trauma histories, difficulties with interpersonal attachment, and general distrust toward social systems often pose barriers to effective treatment engagement for persons with criminal-legal involvement [29, 37–39]. DBT-J therefore employs various targeted engagement strategies to promote effective, sustained participation in DBT-J programming. A trauma-informed approach emphasizes participant choice while simultaneously acknowledging that the VCLI’s experience of navigating systems has limited their choices, consistent with dialectical strategies used in DBT [22]. Many VCLI may feel entrapped or otherwise devoid of choice throughout their navigation of the criminal-legal system. In contrast to this dynamic, DBT-J is presented as completely voluntary, even if being completed as part of a court-issued treatment mandate. VCLI are encouraged to embrace their freedom of choice when deciding whether to enroll in the DBT-J program and whether to continue participation. Should VCLI decide to discontinue participation, they are provided the option of changing their decision until their fourth consecutive group absence, similar to standard DBT.

DBT-J providers also work to tie participation and engagement to VCLI’s pre-existing goals, such as reunification with family, achieving financial independence, and moving past their criminal-legal difficulties. In doing so, VCLI are encouraged to view DBT-J participation as a step in their journey toward building the lives they want for themselves. Correspondingly, providers demonstrate personal investment in VCLI goals, progress, and accomplishments by tying introduced skills and program content to VCLI’s previously expressed goals (e.g., “Could we role play how you might practice this skill during your upcoming court appearance?), checking in about progress toward goals (e.g., “How did that court appearance go last week?”), and celebrating progress and achievements (e.g., “I’m so impressed how you were able to use skills to keep your composure during court last week!”). VCLI are also encouraged to connect with DBT-J providers between session to share progress, achievements, and other updates.

Targeted considerations also encourage appropriate engagement over a virtual platform. Many VCLI may struggle to engage in effective interpersonal behaviors due to several factors, including emotional reactivity, judgmental thinking patterns, interpersonal hostility, and deficits in empathy and impulse control [28, 40, 41]. To promote effective interpersonal behavior, particularly within group-based treatment settings, the first skill introduced within the group therapy portion of DBT-J is “observing limits” – a skill focused on respecting the limits of others and avoiding values-inconsistent behaviors. Where necessary, DBT-J providers also employ contingency management and shaping-based strategies to encourage effective engagement. For example, providers may limit the speaking time of participants when they engage in interpersonally ineffective behaviors (e.g., interrupting others, speaking out of turn) and extend speaking time when participants engage in interpersonally effective behaviors (e.g., staying on topic). Similarly, providers may initially encourage low-engagement participants to join sessions with video camera off to promote consistent attendance before then encouraging attendance with camera on.

Telehealth delivery may present a barrier to engagement for some VCLI, particularly those experiencing financial instability and those with less comfort using technology. To mitigate these barriers, DBT-J providers and staff offer, as needed, formal orientation to the virtual delivery platform, technological troubleshooting assistance, and referral to the VA Digital Device Program, which provides no-cost, internet enabled tablets for accessing telehealth-based services.

DBT-J providers and staff also consistently engage in “friendly harassment” – frequent contact with VCLI both in and out of session. On days of scheduled sessions or assessments, VCLI receive multiple reminders of the impending session using multiple modalities, such as email, phone, and text message. Targeted outreach and shaping strategies are also used with VCLI running late to sessions to promote attendance.

### Need for continued research

Research to date attests to the promise of DBT-J as an intervention for VCLIs. However, it has relied on pre-post designs with modest sample sizes, making it unclear whether observed effects stemmed from participation in DBT-J treatment or extraneous variables, such as the passage of time or receipt of outside services. Continued research using a larger sample and comparison against a control condition is therefore needed to evaluate the potential efficacy of DBT-J.

Further research is also needed to clarify differences in DBT-J treatment response across VCLI subgroups, such as those with severe mental health diagnoses, active substance use disorders, violent offense histories, and/or elevated acute risk for suicide. Such difficulties are commonly considered exclusion criteria for behavioral health programs and many psychotherapy trials, including those provided to justice-involved persons [42–45]. The protocol outlined in the remainder of this manuscript is being used to assess the efficacy of DBT-J and address these limitations of prior research on this intervention.

## Randomized clinical trial

A multisite randomized Stage III clinical trial will be conducted to assess the efficacy of DBT-J. A total of 200 Veterans will be recruited across three regions in the U.S. (VA VISNs 2, 19, and 21) over three years (2024–2026; anticipated date of recruitment completion, June 30, 2026). Random assignment, stratified by region and generated using an electronic random number generator, will be used to assign participating Veterans to DBT-J or a supportive group therapy condition. Assignment will be completed immediately after enrollment and available only to the PI and individuals involved in intervention delivery; assignment is masked from individuals involved in assessment completion. Veterans will receive 16 weeks of assigned intervention followed by a 36-week observation period. To inform evaluations of treatment efficacy, participants will also complete assessments in 8-week intervals during the intervention period beginning at baseline and 12-week intervals during the follow-up period (6 times total). See Table 2. All protocols were developed in accordance with the Declaration of Helsinki and have been approved by the VA Central Institutional Review Board; protocols and analysis plans are pre-registered through clinicaltrials.gov (NCT05974553, registered July 26, 2023). All participants will provide informed consent prior to participation. To preserve participant confidentiality, individual participant data will not be shared with anyone outside of the immediate research team.

**Table 2:**
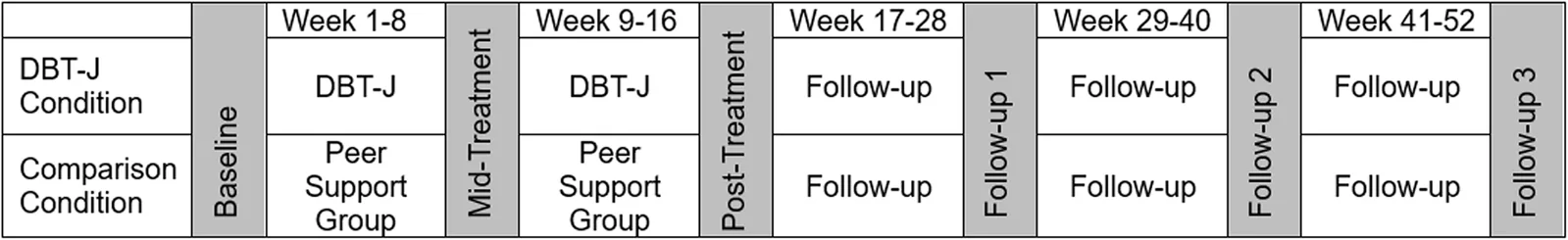
Research design

### Study aims

Study design was guided by the following aims:

1. *Primary Aims*: Assess the superiority of DBT-J over supportive group therapy in (a) decreasing risk of future criminogenic behavior and (b) increasing psychosocial functioning.
2. *Secondary Aim*: Assess the superiority of DBT-J over supportive group therapy in improving secondary treatment targets (i.e., suicide-related thoughts and behaviors, criminogenic thinking, psychopathic personality traits, psychological distress, emotion dysregulation, posttraumatic stress, substance use, case management needs, quality of life, resilience, and criminal recidivism).
3. *Exploratory Aim 1*: Assess for differential efficacy of DBT-J across VCLI subgroups (i.e., violent versus nonviolent most recent offense type, presence/absence of a substance use disorder, and presence/absence of a severe mental illness).
4. *Exploratory Aim 2*: Assess long-term impact of DBT-J participation (versus participation in supportive group therapy) on primary efficacy outcomes.

### Participants

Veterans will be recruited via referral from local mental health providers, VA VJP Specialists, treatment court providers, and local Veteran service organizations and via self-referral through mail-based recruitment. To be considered for inclusion, Veterans must be aged 18 + ; able to provide consent (i.e., have capacity to understand the study’s purpose, risk, and benefits and make an informed decision about participation); and have a recent history of criminal-legal involvement, defined as criminal arrest, order of protection, or incarceration within two years prior to participation and/or supervision by probation or parole at the time of enrollment. Exclusion criteria include limited English proficiency; inability to tolerate group therapy format; concurrent enrollment in another clinical trial; concurrent enrollment in interventions based in the RNR model (e.g., MRT) or DBT; and prior participation in DBT-J. Using similar inclusion/exclusion criteria, prior studies have yielded VCLI samples that are predominantly male with notable heterogeneity in race, ethnicity, age, presenting mental health concerns, and criminal-legal history [20, 35]. To maximize generalizability of results to the heterogeneous population of VCLIs, no inclusion/exclusion criteria related to psychopathology and/or VA eligibility will be used. VCLI experience a range of psychosocial needs [7], and many are not eligible for comprehensive VA care due to discharge status or other barriers [46]. Previous trials have successfully enrolled VCLIs across the psychosocial need spectrum, including those with active suicidal ideation, homelessness, psychotic disorders, and severe substance use [20, 35], suggesting feasibility of this flexibility in inclusion/exclusion criteria.

Consistent with previous DBT-J trials [20, 35], we anticipate a dropout rate of 20% during the intervention period, resulting in a final sample size of approximately 160 at post-treatment. Participants are considered to have dropped out of participation after missing four consecutive group sessions. There are otherwise no attendance requirements for participation.

### Study design

#### Study conditions

Veterans in the DBT-J condition will receive 16 weeks of manualized DBT-J programming, including weekly 1-h group therapy, biweekly 30-min individual case management (20 h of total intervention), routine assessments that guide intervention, and provider participation in a DBT-J consultation team as described above. See Table 3 for schedule of intervention and assessment delivered in the DBT-J condition.

**Table 3:** DBT-J treatment & assessment schedule

Group Skills Module	Week	Skill(s) Introduced	Other
N/A	Pre-Tx	N/A	Assessment
Introduction	1	Observing Limits	
Mindfulness Skills	2	Mindfulness Introduction	Case Management
	3	Wise Mind & Dialectical Thinking	
Emotion Regulation Skills	4	Mindfulness of Self	Case Management
	5	Emotional Schemas & Self- Validation	
	6	Thinking Errors	Case Management
	7	Check the Facts & Opposite Action	
	8	Self-Soothe	Assessment;Case Management
Goal Directedness Skills	9	Values Clarification	
	10	Mindfulness of Goals	Case Management
	11	Problem Solving	
	12	Distress Tolerance & Acceptance	Case Management
Interpersonal Skills	13	Prosocial Assertiveness	
	14	Mindfulness of Others	Case Management
	15	Building & Maintaining a Prosocial Support Network	
Termination	16	Relapse Prevention	Assessment;Case Management

The comparison condition will provide 16 weeks of clinician-facilitated, supportive group therapy. Sessions will occur weekly and last 75 min (20 h of total intervention), allowing for equivalence in total intervention time across conditions. Consistent with the common factors approach to building comparison psychotherapy conditions [47, 48], supportive group sessions will emphasize building alliance between group members and clinicians; clinician expressions of empathy; clear expectations regarding group participation; adaptation to the target population; and providing opportunity to discuss feelings, concerns, and problems. The clinician facilitator will serve to point out common themes and encourage group participation. Sessions will have no preplanned agenda or structured exercises and will not include elements considered specific to DBT-J (i.e., theoretical foundations in RNR, DBT, and/or SDM models, manualized approach, clinician-introduced and/or guided rehearsal of behavioral skills, or provision of individualized case management).

Both conditions will prioritize supporting the needs and experiences of VCLIs, have a closed-group format, and have an enrollment cap of 8 members per group, allowing Veterans to build meaningful relationships with other group members throughout the treatment period. Both will also be delivered via telehealth to ensure program accessibility regardless of participants’ housing status, transportation barriers, or physical location. All interventions will be provided as an adjunctive service, allowing Veterans to continue care with outside providers throughout their participation in the clinical trial. Information about study participation will be documented in VA electronic medical records to inform outside treatment planning and ensure continuity of care. Following completion of the 16-week intervention period, Veterans will continue treatment as usual with their outside providers for the 32-week observation period.

#### Training & fidelity monitoring

Prior to delivering trial interventions, all providers will participate in a comprehensive, 12-h training in the DBT-J and supportive group therapy interventions. Training includes didactic orientation to theoretical basis and intervention strategies of each intervention and role play exercises to develop and strengthen relevant clinical competencies. Given the unique needs of VCLI and previously documented barriers to implementing forensic-oriented services in VA settings, training also actively identifies and combats commonly held stigmas toward criminal-legal involvement, antisocial behavior, and personality disorders and emphasizes therapeutic engagement strategies, suicide risk assessment and management protocols, and strategies for managing group dynamics over a virtual platform. All research staff involved in provision of study assessments similarly complete standardized trainings, including didactics, role play exercises, and coding of training tapes, prior to delivering any study assessments.

A randomly selected 20% of group (DBT-J and supportive group therapy conditions) and case management (DBT-J condition) sessions will be recorded and reviewed by an independent rater to assess fidelity to treatment protocols. Ratings will be guided by objective scales designed to assess core features of program structure, content, and treatment principles. Providers must maintain an average of 4 out of 5 (some minor violations to adherence) or above on the fidelity scale to demonstrate adequate fidelity. Providers receiving scores of 3 or lower will receive rehabilitative supervision and constant fidelity monitoring until fidelity is re-established.

### Assessment of treatment efficacy

#### Participant characteristics

Demographics assessment will include participant self-reported age, sex, gender, race, ethnicity, sexual orientation, socioeconomic status, employment status, disability status, educational attainment, relationship status, and religious affiliation. Chart and records review, including review of Veterans’ VA medical charts and official criminal history records, will be used to assess history of suicide-related behaviors and history of criminal-legal involvement.

The Level of Service / Case Management Inventory (LS/CMI; [31] will be used to assess risk of future criminal behavior. The LS/CMI is a semi-structured interview assessment based on the RNR model of criminal behavior. It has been widely validated, with the total score as a good predictor of later criminal behavior [49]. Scores are interpreted through comparison against normative data of over 150,000 North American offenders [31].

The Columbia Suicide Severity Rating Scale (C-SSRS; [50]) will be used to assess history of suicide-related behaviors, including suicidal thoughts and behaviors. The C-SSRS is a semi-structured interview that assesses prevalence, recency, and nature of prior suicide-related behaviors. It has been widely used in both VA- and civilian-based settings and demonstrates strong inter-rater reliability and validity in Veteran samples [51, 52].

Mental health diagnosis will be assessed using the Quick Structured Clinical Interview for DSM-5 (QuickSCID-5; [53], and the Structured Interview for DSM-IV Personality (SIDP-IV; [54]. Each is a semi-structured interview to assess for mental health conditions according to established diagnostic criteria.

#### Primary efficacy outcomes

To inform primary aim analyses, participants will complete measures of risk for criminal behavior and psychosocial functioning at all assessment timepoints. Risk for criminal behavior will be assessed using the LS/CMI, described above. Psychosocial functioning will be assessed using the Inventory of Psychosocial Functioning (IPF; [32], an 80-item, self-report instrument measuring functional impairments in romantic relationships, family relationships, work, friendships and socializing, parenting, education, and self-care. Scores are interpreted through comparison to normative data of Veterans [55]. Previous research suggests the IPF to have strong construct validity, concurrent validity, internal reliability, and test–retest reliability [55].

#### Secondary efficacy outcomes

To measure secondary outcomes, participants will complete a range of self-report measures, including the Beck Scale for Suicidal Ideation (BSS; [33], Screen for Nonsuicidal Self-Injury (SNSI; [56], The Measure of Criminogenic Thinking Styles (MOCTS; [57], Triarchic Psychopathy Measure (TriPM; [58]), Patient Health Questionnaire-9 (PHQ-9; [59], PTSD Checklist for DSM-5 (PCL-5; [60], Difficulties in Emotion Regulation-18 (DERS-18; [61], Alcohol Use Disorders Identification Test (AUDIT; [62]), Drug Abuse Screening Test (DAST-10; [63], DBT-J Case Management Checklist [64], Quality of Life Enjoyment and Satisfaction Questionnaire—Short Form (Q-LES-Q-SF; [65]), and Connor-Davidson Resilience Scale (CD-RISC; [66]). To complement these assessments, a chart and records review will be completed by an independent rater to assess for nature and total duration of VA-based mental health care received, presence and frequency of suicide-related behavior documented in VA electronic medical records, and presence and frequency of criminal recidivism (defined as new arrest, violation, order of protection, or incarceration) occurring during the study period.

#### Assessment protocol

Each of the six assessment timepoints will include a combination of semi-structured interviews completed via video call (including LS/CMI at all timepoints, C-SSRS at baseline and final follow-up, and QuickSCID and SID-P-IV at baseline) and self-report questionnaires self-administered via Qualtrics. See Table 4. To minimize bias in assessment, all interviews will be completed by evaluators who are blind to participant condition. Veterans will receive monetary compensation ($75) for each completed assessment; a completion bonus ($75) will also be provided at the end of study participation to participants who complete all assessment timepoints to encourage retention throughout the follow-up period. A random 20% of interviews will be recorded and reviewed by a second rater to assess fidelity to assessment administration protocols and determine inter-rater reliability.

**Table 4:** Projected assessment schedule

Assessment	Construct	Baseline	8 weeks	16 weeks	28 weeks	40 weeks	52 weeks
Participant Characteristics							
Demographic Assessment	Demographic background	X					X
Chart & Records Review	Psychiatric treatment history, Military service history, Suicide-related behavior history, History of criminal-legal involvement						X*
QuickSCID / SID-P-IV	Mental health diagnoses	X					
C-SSRS	Suicide-related behavior history	X					X*
Primary Outcomes							
LS/CMI	Risk for criminal behavior, History of criminal-legal involvement	X	X	X	X	X	X
IPF	Psychosocial functioning	X	X	X	X	X	X
Secondary Outcomes							
BSS	Risk for suicide-related behavior	X	X	X	X	X	X
SNSI	Non-suicidal self-injury	X		X			X
DERS-18	Emotion regulation difficulties	X	X	X	X	X	X
TriPM	Psychopathic personality traits	X		X			X
MOCTS	Criminogenic thinking style	X		X			X
PHQ-9	Psychological distress	X	X	X	X	X	X
AUDIT	Alcohol use	X	X	X	X	X	X
DAST-10	Non-alcohol substance use	X	X	X	X	X	X
DBT-J CM Checklist	Case management needs	X	X	X	X	X	X
Q-LES-Q-SF	Quality of life	X	X	X	X	X	X
CD-RISC	Resilience	X	X	X	X	X	X
PCL-5	PTSD symptom severity	X		X			X

^*^Or at time of study termination

A data safety monitoring board (DSMB) comprised of research psychologists with experience in high-risk Veteran populations and intervention development research will aid in monitoring ongoing data collection to evaluate for potentially adverse effects of study participation. Collected data will be shared with the DSMB at least quarterly or within 48 h of an adverse event, whichever occurs first.

### Data analysis plan

Analyses will employ an alpha of 0.05, use two-tailed tests, and be completed using SPSS and/or the R Computing Environment. Preliminary analyses will include computing descriptive statistics and inspecting data to determine whether data transformations are needed, statistical assumptions are met, and psychometric properties of each scale are sound. Multiple imputation will be used for transient missing data. Because participants who dropout of the protocol may be qualitatively distinct from protocol completers (e.g., due to differences in motivation, legal requirements, etc.), analyses will also include a sensitivity analysis by comparing the full “intent-to-treat” sample against protocol completers. Potential confounders, including duration of outside treatment services received during the intervention period, will be compared across conditions and included as covariates in subsequent analyses if significantly related to study outcomes.

Primary and secondary analyses will be investigated using analyses of covariance to compare participants in the DBT-J and supportive group conditions on primary and secondary continuous treatment targets at post-treatment after controlling for baseline scores. Poisson regression with robust error variance will similarly be used to compare DBT-J and comparison conditions on dichotomous secondary treatment targets (suicide attempt during study period and criminal recidivism during study period). The DBT-J condition will be considered superior to the supportive group condition if participants in the DBT-J condition demonstrate statistically superior scores on either primary treatment target at post-treatment. The DBT-J intervention will be considered efficacious if results suggest (a) superiority to the supportive group condition and (b) clinically significant change in primary efficacy outcomes from baseline to post-treatment among DBT-J participants [67].

To support exploratory analyses comparing efficacy of DBT-J programming across VCLI subgroups, DBT-J participants will be coded according to most recent offense type (violent or nonviolent), presence of a substance use disorder, and presence of a severe mental illness (i.e., mood or psychotic disorder). Multivariate analyses of covariance will be used with subgroup membership included as predictor variables, LS/CMI and IPF post-treatment scores included as dependent variables, and LS/CMI and IPF baseline scores included as covariates.

Lastly, exploratory analyses will examine potential long-term consequences of participation in DBT-J versus supportive group therapy on primary efficacy outcomes. Two repeated-measures analyses of variance will be used with condition included as the independent variable and LS/CMI or IPF total scores at each assessment timepoint included as dependent variables.

#### Justification of sample size

A priori power analysis was used to determine an appropriate sample size to support primary aim analyses. Informed by results of previously collected pilot data [20, 35], we anticipated at least a moderately-sized difference across conditions in primary efficacy outcomes at post-treatment. Power analysis results suggested at least 80 participants per condition (160 total) are needed to achieve 80% power to reject the null hypothesis of equal means when the population mean difference is *d* = 0.50 and α = 0.025 (to adjust for multiple comparisons). After adjusting for an anticipated dropout rate of 20%, 100 participants per condition (200 total) are required to achieve necessary statistical power.

### Anticipated limitations

Due to under-representation of women in VCLI samples [68], we anticipate difficulties obtaining a sample that will support analyses surrounding potential sex differences. To offset this potential limitation, recruitment efforts will attempt to over-sample for women through specific outreach to VA providers specializing in the care of female Veterans. Further, although previous research suggests low attrition rates from DBT-J, ample research attests to the tendency for Veterans to dropout of standard behavioral health services prematurely [69]. It is therefore possible that attrition will be higher than expected during the intervention period, particularly for VCLIs assigned to the comparison condition. The study design also does not allow for explicit comparisons of DBT-J against current standard of care for VCLIs. Should results suggest DBT-J to be an efficacious treatment, continued research should explore this comparison.

## Discussion

Through reduction of criminogenic and suicide risk and resolution of case management needs, DBT-J has the potential to attenuate psychiatric symptoms, improve quality of life, and bolster psychosocial functioning among VCLIs. The program builds on the strengths of existing outreach to VCLI through Veterans Justice Programs and fosters direct access for these high-risk, high-need Veterans to responsive resources they might not otherwise receive by traditional VA services. Prior DBT-J participants have often described considerable difficulties in accessing services due to stigma around criminal involvement, complexities of presenting needs, and institutional mistrust. However, through DBT-J, VCLIs are engaged in meaningful treatment and connected to needed services, thereby promoting effective rehabilitation.

The DBT-J intervention and this clinical trial protocol have been specifically tailored to meet the needs of a population at concurrent risk for both suicide and criminal-legal involvement. Although DBT-J was developed to capitalize on supports and structures offered through the VA system (e.g., Veterans Justice Programs, streamlined referral procedures), many considerations and intervention strategies involved in DBT-J development and evaluation may readily apply to other contexts targeting justice-involved persons at risk for suicide. For example, although DBT-based programming is routinely implemented in forensic settings [25, 38], specific adaptations to the DBT intervention to facilitate such implementation are rarely noted explicitly in the literature. The DBT-J program outlined here may serve as a model for forensic-DBT programs by highlighting the nature of and rationale for included adaptations, particularly the restructuring of skills training content to draw on both DBT- and RNR-based intervention strategies.

The DBT-J program also suggests heavy use of engagement strategies – particularly frequent contact and demonstrations of personal investment in participant-identified goals – appears useful in promoting service engagement and retention in this often difficult-to-engage population. The combination of formal risk assessment and ongoing risk monitoring throughout the course of program delivery also facilitates a time-efficient, person-centered approach to suicide risk assessment and management that fosters collaboration between providers and program participants while minimizing the need for repeated administration of lengthy assessment measures. Further, the DBT-J intervention highlights the importance of prioritizing cultural responsivity throughout program development and delivery; such responsivity appears key to improving accessibility and perceived relevance of intervention content and delivery. Application of such considerations is encouraged in both research and practice with justice-involved persons at risk for suicide.

Results of this research will inform broader efforts to improve quality of care provided to VCLIs both within and outside of the VA. Results will be disseminated via reporting in trial registry, peer-reviewed publication, presentation at local and national conferences, and plain-language summary shared with participants and VA operational partners. Should results suggest DBT-J to be efficacious, longer-term objectives for this line of research include investigations of scalability, sustainability, and cost-effectiveness of program delivery; dissemination of DBT-J to VA providers; implementation of DBT-J into VA services and other services specific to VCLIs (e.g., Veterans treatment court); and investigations into therapeutic mechanisms of change. By contrast, if results misalign with hypotheses, results may be used to inform improvements to the DBT-J protocol and/or development of more efficacious interventions for VCLIs.

## Supplementary Information

Additional file 1.

## Data Availability

No datasets were generated or analysed during the current study.
